# Animal-Human Health Interface and community based surveillance in Vietnam-a strategy under Mekong Basin Disease Surveillance Cooperation (MBDS)

**Published:** 2011-01-10

**Authors:** Nguyen Dang Vung

**Affiliations:** 1 Department of Demography, Hanoi Medical University, Hanoi 10000, Vietnam

## 

In recent years, zoonoses have been increasing. 75 % of emerging diseases are zoonoses such as SARS, Influenza A/H5N1, Rabies, Streptococcus suis, Nipah virus, etc. in Vietnam, ministries of health and Agriculture ate not collaborating closely enough to control zoonotic disease, information sharing between two ministries does not currently occur regularly and is generally only done when requested. Community based surveillance has not been done systematically and effective.

MBDS cooperation (including Cambodia, China, Laos, Myanmar, Thailand and Vietnam) was established in 1999 with financial support from Rockefeller Foundation. Its goal is to strengthen national and subregional collaboration on the interface between human and animal health, i.e., between Ministries of Health and Agriculture, to rapidly and effectively detect and control communicable diseases that are spread by poultry and animals and may affect humans. The 3^rd^ phase of MBDS (2008-2010) focuses on 7 strategies including cross-border collaboration, animal-human health interface, capacity building, information communication technology development, laboratory development, risk communication and policy research.

After two years of implementation MBDS Vietnam, we have achieved effective results. The animal-human health departments sit together to develop interministerial circulars, for information sharing and collaboration in the prevention and control of zoonotic outbreak. Every year, two workshops were organized for animal and health sectors at national and provincial and district levels to share information, experiences and develop the collaboration mechanism. Animal and human health sectors have conducted 4 joint outbreak investigations on A/H5N1 in 2008 and 2009. Cross-border meetings have been organized between provinces of Vietnam with provinces of Cambodia, Lao PDR and China. Two Table Top Exercises on A/H5N1 control between animal and health sectors have been implemented (in Lai Chau and An Giang provinces). We have developed the model of community based surveillance in Quang Tri and An Giang provinces. Every year, MBDS organized Regional Forum for member countries to share information, experiences in outbreak surveillance, investigation, prevention and control.

